# Evolution, Mechanisms, and Applications of Intein-mediated Protein Splicing

**DOI:** 10.1074/jbc.R114.570531

**Published:** 2014-04-02

**Authors:** Francine B. Perler, Norma M. Allewell

**Affiliations:** From ‡ New England Biolabs, Ipswich, Massachusetts 01938 and; the § Department of Cell Biology and Molecular Genetics, University of Maryland, College Park, Maryland 20742

**Keywords:** Enzyme Mechanisms, Protein Evolution, Protein Engineering, Protein Processing, Protein Structure, Hedgehog, Biotechnology

## Abstract

Intein-mediated protein splicing raises questions and creates opportunities in many scientific areas. Evolutionary biologists question whether inteins are primordial enzymes or simply selfish elements, whereas biochemists seek to understand how inteins work. Synthetic chemists exploit inteins in the semisynthesis of proteins with or without nonribosomal modifications, whereas biotechnologists use modified inteins in an ever increasing variety of applications. The four minireviews in this series explore these themes. The first minireview focuses on the evolution and biological function of inteins, whereas the second describes the mechanisms that underlie the remarkable ability of inteins to perform complex sets of choreographed enzymatic reactions. The third explores the relationship between the three-dimensional structure and dynamics of inteins and their biochemical capabilities. The fourth describes intein applications that have moved beyond simple technology development to utilizing inteins in more sophisticated applications, such as biosensors for identifying ligands of human hormone receptors or improved methods of biofuel and plant-based sugar production.

## Introduction

Inteins are proteins that have the remarkable ability both to excise themselves from precursor proteins and to ligate their flanking polypeptides to yield a mature host protein (extein) and a stable intein. They have captured the attention of diverse groups of scientists who wonder how they work, where they came from, and how they can be used. Although progress has occurred in each of these areas, there is still much to be learned.

Intein evolution, biological distribution, and biological function are the themes of the first minireview by Novikova *et al.* (1). Inteins share a structural fold with the essential metazoan Hedgehog signaling protein autoprocessing domain, suggesting that inteins evolved from ancient proteins that predate the separation of prokaryotes and eukaryotes (2, 3). Many modern intein genes are mobile genetic elements encoding intein proteins with a homing endonuclease domain that is able cleave DNA at the site at which the intein coding sequence is inserted. The location of inteins within conserved extein motifs has helped to ensure their survival because imprecise intein deletions would inactivate the extein protein and reduce the viability of the host. It also enhances horizontal transfer by decreasing DNA sequence variability at the homing endonuclease recognition site. Although modern mobile intein genes represent selfish DNA, Novikova *et al.* suggest that conditional protein splicing may occur in natural systems and that the role of inteins in present day organisms may be to regulate protein function. The search for an example of naturally controlled splicing in a native organism is ongoing.

Although the basic class 1 intein splicing pathway was determined 20 years ago, the details of the splicing mechanism are just beginning to be elucidated. Inteins are single turnover enzymes that use all of the strategies that enzymes can muster to achieve catalysis under physiological conditions in a reasonable time frame. The second minireview in this series, by Mills *et al.* (4), focuses on the rich variety of splicing mechanisms that utilize different combinations of amino acids in the catalytic mechanism of each different intein. Many aspects of splicing have been identified through a combination of mutation, comparative sequence analysis, structural approaches, and molecular dynamics simulations. However, major mechanistic questions remain, including control of the steps in the splicing reaction, the effects of exteins, and the full repertoire of strategies that diverse inteins use to achieve catalysis. Finally, the question of whether insights gained from studying one intein can be applied to all inteins or only to inteins with a similar set of nucleophiles and assisting amino acids remains to be determined.

The third minireview in this series, by Eryilmaz *et al.* (3), explores the mechanism of protein splicing from a structural and molecular dynamics perspective and reports that, in native systems studied to date, splicing appears to occur as soon as the intein folds. This minireview also discusses the theme of *trans-*splicing with split precursors and its implications for nucleation of folding in all inteins. It further examines the possibility that inteins can mediate domain shuffling by *trans*-splicing, a possibility that would promote evolution while maintaining the genome.

The last minireview in this series, by Wood and Camarero (5), describes a very full intein toolbox. Early uses of inteins include protein purification and the ability to generate a C-terminal thioester on a target protein to serve as a building block for various tagging, binding, and semisynthetic applications. Intein technology has moved past these initial applications into more complex second-generation applications, such as intein-based biosensors, redox state detectors, conditional protein activation, gene delivery components, and control of transgene function. Applications discussed in this minireview include intein biosensors that have been developed to detect ligands of human hormone receptors and the use of inteins to prevent the transmission of transgenes from genetically modified organisms. A third area of intensive study is conditional protein splicing. For example, temperature-dependent splicing was used to regulate a xylanase introduced into corn so that it is inactive until post-harvesting heat treatment induces splicing, which in turn activates it and enables “self-processing” of the cellulosic biomass.

Collectively, these minireviews summarize our current understanding of protein splicing, the questions yet to be answered, and the potential of intein applications. Intein mechanisms provide insight into how enzymes adapt to mutations in a system that is more amenable to change because rapid turnover and substrate binding are not limiting. Recent advances in the diversity and relevance of intein applications reflect a maturation of their potential as tools for modifying, synthesizing, and controlling protein function.
